# Autism in Preschoolers: Does Individual Clinician's First Visit Diagnosis Agree with Final Comprehensive Diagnosis?

**DOI:** 10.1155/2013/716267

**Published:** 2013-09-04

**Authors:** Gunilla Westman Andersson, Carmela Miniscalco, Christopher Gillberg

**Affiliations:** Gillberg Neuropsychiatry Centre, Sahlgrenska Academy, University of Gothenburg, Kungsgatan 12, 411 19 Gothenburg, Sweden

## Abstract

Comprehensive clinical diagnosis based on all available information is considered the “gold standard” in autism spectrum disorders (ASD). We examined agreement across independent assessments (clinical judgment) of 34 young children (age 24–46 months) with suspected ASD, assessed by a multidisciplinary team, and final comprehensive clinical diagnosis. Agreement across settings and between each clinician's assessment and final diagnosis was moderate. The poorest fit was found at assessment in connection with psychological evaluation and the best with preschool observation and parent interview. Some individual clinicians had good and others had poor fit with final diagnosis. Disagreement across assessments was pronounced for girls. The findings suggest that multidisciplinary assessments remain important and that comprehensive clinical diagnosis should still be regarded as the gold standard in ASD.

## 1. Introduction

In an international perspective, there is now a strong focus on autism spectrum disorders (ASD) and the importance of its early diagnosis in toddlers and preschool children. Early identification of problems and early intervention are important for the child's positive development [1, 2]. ASD is usually congenital and involves early childhood symptomatic restrictions in reciprocal social communication and stereotyped behaviors [3]. It occurs at different levels of general intelligence, with a prevalence of about one percent of the general population, about 0,8% in preschool children [4], and a much raised male : female ratio [5–7]. In a newly published Swedish study [8], preschool girls and boys were recruited through population screening, assessed for suspected ASD, and matched by chronological and developmental age. No significant differences were found in their developmental profiles, a result contrary to earlier studies [9]. Therefore, there is still a need for further research in gender differences in ASD, to develop diagnostic instruments that are appropriate for both girls and boys. 

The definitions for diagnosis in the field of ASD—or Pervasive Developmental Disorders (PDD) as they are referred to in most diagnostic manuals—are currently based on the International Classification of Diseases, Tenth Revision (ICD-10) [10] or the Diagnostic and Statistical Manual of Mental Disorders, Fourth Edition (DSM-IV) [11]. The DSM-5 (http://www.dsm5.org) introduces the concept of an autism “spectrum”, and this is the first time that what was previously considered a distinct diagnostic *category* will be referred to as *a spectrum of disorders*. Clinical judgment by experienced clinicians is seen as crucial in the diagnostic process and considered the gold standard for diagnosis [12]. Diagnosing young children is often a complex process, and the early identification of core symptoms is very important. Clinicians need to have good knowledge regarding common developmental disorders and medical/psychiatric conditions in young children and of normal “age-typical” child development [3, 6, 13]. Symptoms of ASD in 2-year-old children may differ (even markedly) from symptoms identified at the age of 4 or 5 years. For instance, in a 2-year-old, particular types of repetitive and stereotyped behaviors and interests may be less obvious than a few years later, whereas other behaviors and interests may show the opposite trajectory. Even if standardized assessments are very important, clinical experience/judgment is suggested to be more reliable regarding young preschool children [14]. 

There is a high degree of phenotypical variation in ASD, and no single instrument or algorithm, for example, Diagnostic Interview for Social and COmmunication disorders (DISCO) [15] or Autism Diagnostic Observation Schedule (ADOS) [16], can be used to “finalize” the diagnosis, particularly since there are many other factors, including chronological age, overall developmental/cognitive level, and “comorbidity,” that need to be taken into account [17]. 

Certain difficulties, including problems pertaining to joint attention, have been suggested to be strong predictors of ASD. However, there are many children with ASD who do not fail to respond to joint attention and even those who do appear to initiate it [18]. This means that joint attention tests in themselves cannot be used as “diagnostic arbiters” for ASD. In order to collect even the minimum (=sufficient) amount of information about the child, needed to arrive at an appropriate clinical diagnosis, multidisciplinary diagnostic assessments can be very important. Interviews with parents or other caregivers and free-field structured observation of the child in social settings, in addition to formal test instruments, are suggested to be very important in the diagnostic process, not least in young children. It is also generally agreed that clinical assessments for ASD should be done by clinicians with extensive experience [17].

ASD is one of the diagnostic categories subsumed under the umbrella concept of Early Symptomatic Syndromes Eliciting Neurodevelopmental Clinical Examinations (ESSENCE). ESSENCE refers to developmental problems, presenting as “syndromes” in clinical settings before 5(-6) years of age [6]. Symptoms are classifiable as stemming from problems in terms of (a) general development, (b) communication-language, (c) social inter-relatedness, (d) motor coordination, (e) attention, (f) activity, (g) “general” behavior; (h) mood, and/or (i) sleep. If problems in one of these fields have been identified, it has become commonplace to refer the child to a specialist within a specific field, when in reality the child would have required a more multidisciplinary approach to assessment and intervention. If the child shows major problems in at least one of the problems listed before 5(-6) years of age, there is a high risk of major problems in the same or overlapping area several years later. Often, these “overlapping” problems nowadays go unnoticed, undiagnosed, and untreated for years. However, evidence exists to indicate that, whenever a diagnosis of ASD is made, there is a need to also be on the look-out for attention-deficit/hyperactivity disorder (ADHD), tic disorders, intellectual developmental disorder (IDD) [19], speech and language disorder, epilepsy and other “medical disorders,” and so forth, at the same time [6, 20]. There is no good reason to wait for the identification of the whole range of non-ASD problems that exist in virtually all young children with ASD at the earliest possible age. 

All of this would suggest an even stronger rationale for a multidisciplinary assessment in all children suspected of suffering from ASD at a young age. Conversely, however, due to the high prevalence of suspected ASD (about one percent of the general population of toddlers), there is a need to find out which “autism specific” assessments carry the highest potential of predicting the “true positive” gold standard clinical ASD caseness; is it the doctor's medical examination, the psychologist's testing, the autism observation assessment at the clinic, the detailed parent diagnostic interview, or free-field observation in the child's preschool? Or are we still in need for multidisciplinary assessment?

Taking these partly opposing needs (multidisciplinary versus focused “lean”) into account, we designed a study of ASD in preschoolers, nested in the context of an ASD general population cohort, looking at agreement across a variety of separate autism focused assessments and the ability of each of these to predict final conjoint comprehensive clinical ASD diagnosis.

### 1.1. Aim of the Study

The aims of the present study were to evaluate (1) the agreement between different, independent, expert clinicians' first clinical impression of young children with suspected ASD and (2) the agreement between their assessment and the final clinical comprehensive ASD diagnosis. We argued that getting a handle on these issues would have the potential of providing guidance regarding the extent of assessment needed in young children with suspected ASD, and of the status of clinical global impressions vis-à-vis certain formal tests.

## 2. Methods

This is a substudy of the larger general population AUtism Detection and Intervention in Early life project (AUDIE), at the Child Neuropsychiatry Clinic (CNC), a study performed in collaboration with Child Health Care Services and Autism Habilitation Centres in Gothenburg [7]. The overall aim of the AUDIE project is to (a) identify all 0–3-year-old children with symptoms of ASD and other developmental disorders in the general population, (b) provide comprehensive clinical assessment and diagnosis without delay, and (c) offer early intervention without delay. All Gothenburg children are screened for language/communication problems and ASD by specially trained well-baby nurses in well-baby clinics at 2.5 years of age and earlier. All children screening positive at 2.5 years or raising suspicion of ASD at any other age below 4 years are referred for ASD in-depth assessments to the CNC. All cases diagnosed with ASD are then referred to the Autism Habilitation Centers for intervention.

### 2.1. Participants

Thirty-four children (6 girls, 28 boys), with an age range of 24–46 months (mean age 34 months, mean Griffiths' developmental quotient (DQ) 82, range 33–128), participated in the study. They had all screened positive for ASD and had been referred for suspected ASD to the CNC, where they underwent comprehensive neuropsychiatric assessment, comprising six different assessment settings (and 12 different assessors representing four different types of profession; see below). All 34 children were seen during a limited time period. Forty-two children had originally been targeted, but eight had to be excluded because data from three or more of the six assessments were missing. No divisions by ethnicity or socioeconomic status were made in this study. 

### 2.2. Assessments and Test Instruments Used

#### 2.2.1. Medical-Psychiatric Neurological Examination by Physician

One of four physicians met the child and the parents at the CNC, observing and interacting with the child in the room and obtaining a medical/developmental/psychiatric history from one or both parents. A neurological examination/brief neuropsychiatric observation assessment of the child was also made. This examination took about 1.5 hour.

#### 2.2.2. Griffiths' Developmental Scales Assessment by Psychologist

One of four psychologists administered the Griffiths' developmental scales I and II [21] which measure the rate of development for children from birth to eight years of age. It consists of six subscales: (a) gross motor; (b) personal-social; (c) hearing-speech; (d) eye-hand coordination; (e) performance and (f) practical reasoning. The combined result of the subscales provides a developmental quotient (DQ). The test took about 45–60 minutes to be completed. 

#### 2.2.3. Language Assessment by Speech and Language Pathologist

One of two speech and language pathologists (SLP) performed the language assessment. Language comprehension was tested with the Reynell Developmental Language Scales III (RDLS) [22], where the Swedish version has norms for 2.0–4.11-year-old [23]. The RDLS contains 62 test items, sorted into 10 different domains, from comprehension of single words to sentences of increasing complexity/difficulty. Expressive language was measured, whenever possible, with formal language tests, and spontaneous speech was transcribed. The SLP assessment took about one hour to be completed.

#### 2.2.4. DISCO by Physician or Psychologist

One physician or one of two psychologists made a parent interview, using the Diagnostic Interview for Social and COmmunication disorders (DISCO) [15], which is a semistructured interview, designed for eliciting systematic information regarding development and behaviors from the start of life until current time, so as to allow well-founded classification of ASD in accordance with a variety of diagnostic systems [24]. In our study at least one of the child's parents participated in the interview. It took 2–4 hours to be completed.

#### 2.2.5. ADOS Assessment by Education Specialist and Psychologist

One of two education specialists plus one of four psychologists administered the Autism Diagnostic Observation Schedule (ADOS) [16]. The ADOS is a standardized, semistructured play-based assessment of communication, reciprocal social interaction, play, and behavior. There are four modules, based on expressive language level. One test manager interacted with the child, and one other professional observed the child during the test. Immediately after the ADOS, both professionals jointly scored the child's performance according to the manual, which has an algorithm for ASD diagnosis cutoff. The ADOS took about 40–50 minutes of the child's time to be performed.

#### 2.2.6. Preschool Observation/Observation at Home by Education Specialist

One of two education specialists from the assessment team observed the child in group activities and free play in accordance with a protocol based on the symptom areas covered by the ADOS. The classrooms were designed for typically developing children, and the number of children in the groups ranged 15–30 children. During the observation, the preschool teachers were with the children as they normally would in everyday situations. The observation took about 45–60 minutes to be performed and was followed by an interview with the teachers regarding the child's abilities and behavior in different situations in preschool, meaning that the total time for the education specialist was on average 2 hours. Eight of the children in the study did not attend a preschool, so the education specialist observed the child in the home for about one hour, with one or more family members around. 

### 2.3. Diagnostic Process

In addition to the above-described instruments, the Vineland Adaptive Behavior Scales [25] (performed by the psychologist) and the CGAS [26] (jointly made by the assessment team) were used in the diagnostic process in all cases. However, these two instruments were not included in this study and were made *after* the individual clinician's first clinical assessments had been documented.

To avoid bias, all six assessments were performed by the different professionals working independently of each other, and this required specific logistics. Under “ordinary” clinical diagnostic conditions, the same professional might well perform both a preschool observation and the ADOS at the clinic, meaning that fewer individual members would have to be included in the assessment team. Here, we had to involve several different clinicians, in order to guarantee blindness across different assessment ratings. The results from each of the six assessments were included on the basis of assigning a final comprehensive conjoint clinical diagnosis. The clinicians remained blind to all other assessment results until the consensus diagnostic case conference, which was held after the completion of all assessments as listed, approximately 4–6 weeks after the first assessment. On the basis of all available information, the assessment team made consensus clinical diagnoses according to the DSM-IV criteria for disorders first evident in childhood or adolescence and CGAS ratings. 

The number of clinical examiners involved in the study was four psychologists, four physicians, two SLPs, and two education specialists. All clinicians except for one had worked in the field >10 years. 

### 2.4. Procedure

In order to avoid bias, different examiners performed all the assessments independently of each other and were blind to any other prior information obtained by other clinicians. The examiner knew only the child's name, age, and gender and the fact that the child had screened positive for ASD at a well-baby clinic check-up. Prior to start of the data collection, the examiners were given oral and written instructions. A separate coding sheet for each examiner's independent assessment of ASD [(1) ASD; (2) ASD probable or possible; or (3) no ASD] was completed at the end of the child's first visit to that examiner. On the coding sheet, there was also a box for notes regarding any other comments the examiner might want to make about the child's problem. The completion of the coding sheet was done before any summaries of formal assessments were made, such as before scoring the ADOS, the DISCO, or Griffiths'. The coding sheet was then put in an envelope, sealed, stored, and opened only after the full diagnostic process had been completed. In addition, the clinicians were instructed not to reveal any information about their stored clinical impression to anybody else (including child, parent, teacher, or any other clinician).

### 2.5. Statistical Methods

Sensitivity and specificity with 95% confidence interval were analyzed for each individual rater with final clinical diagnosis as the golden standard. Agreement across individual assessors and final clinical diagnosis was analyzed by percent agreement and the weighted kappa statistic with 95% confidence interval. Systematic differences across assessors and clinical diagnoses were analyzed using sign test. All significance tests were two-sided and conducted at the 0.05 significance level.

### 2.6. Ethics

This study was approved by the Human Ethics Committee of the Medical Faculty at the University of Gothenburg, Sweden. Informed consent was obtained from at least one of the parents/responsible carers for each patient.

## 3. Results

Twenty-five of the children were clinically comprehensively diagnosed with ASD (16 autistic disorder, 9 pervasive developmental disorder not otherwise specified (PDDNOS)/atypical autism). Five children had autistic traits (1–4 symptoms of DSM-IV autistic disorder), and 4 children had no ASD/no autistic traits. 

### 3.1. Assessment Setting: ASD Codes versus Final ASD/No ASD Diagnosis

The main results are presented in Table 1. The sensitivity versus final clinical diagnosis was the highest for DISCO (0.74), lowest for DQ (0.40) and all other assessments between 0.60 to 0.86. The specificity was much higher, over 0.89 for all raters except for language. Corresponding 95% confidence intervals were rather wide due to the small number of subjects. The DQ assessment setting (Griffiths' testing) yielded the poorest agreement (47%), weighted kappa 0.28 with comprehensive clinical diagnosis, and also showed systematically less ASD than the clinical diagnosis (*P* = 0.007). For all other assessments the percent agreement was between 58% and 68% and weighted kappa between 0.33 and 0.43. 

The DQ assessors “underestimated” almost half (44%) of the children in terms of diagnostic “level” in relation to clinical diagnosis (final clinical diagnosis showed more ASD), whereas the parent interviewers (DISCO) “underestimated” a much smaller proportion (22%) in this respect. In contrast, the DQ assessors “overestimated” only 9% and the parent interviewers 19%.

The number of girls in the study was low, and gender differences were not statistically analyzed due to this. Nevertheless, we consider it to be of interest that the DQ assessors failed to agree with the final clinical diagnosis in all six girls participating in the study. The ADOS assessors agreed in 1/6 (17%), the SLP in 2/5 (40%), the child psychiatrist/neurologist in 3/6 (50%), the educator specialist in 4/6 (67%), and the parent interviewer (DISCO) in 3/4 (75%). All the girls were clinically comprehensively diagnosed with ASD. 

### 3.2. Individual Assessors' Codes versus Final ASD/No ASD Diagnosis

We found a very considerable degree of variability as regards agreement between individual assessor diagnostic codes and final diagnosis, both between and within different professional categories (Table 2). Of the 12 clinical assessors who had assessed five or more children, two of them had more than 80% agreement with final diagnosis and five had less than 50% agreement. 

In addition, we found that the algorithm diagnosis in the ADOS differed from the consensus clinical diagnosis in 7/34 cases (4/7 “less ASD,” 3/7 “more ASD”). For the DISCO it differed in 8/34 cases (1/8 “less ASD,” 7/8 “more ASD”). 

## 4. Discussion

This study found only moderate agreement between “blind” clinical assessors' individual preliminary diagnosis and the final comprehensive conjoint clinical diagnosis based on all available evidence elicited at the various clinical evaluations. Also, agreement across types/classes of raters was not perfect. A tendency for “best fit” with final conjoint clinical (“gold standard”) was found using assessment made immediately after preschool/home free-field observation of the child, and the poorest was that of assessment of clinical diagnostic status made in connection with highly structured DQ assessment. On the other hand the low reliability obtained in spite of the comprehensive assessment performed, casts doubts on the feasibility and usability of the diagnosis of ASD in toddlers and may indicate the need for a broader diagnosis before the age of 4 (developmental disorder) in agreement with ESSENCE [6]. In doubtful cases that should be confirmed at a later stage.

When interpreting these results, it is important to consider how the environmental assessment conditions vary. The psychologist's primary role was to evaluate the child's developmental level and basic abilities. In order to be able to do this, the assessment situation had to be more structured than everyday situations, meaning fewer items of distraction, fewer people in the room, and so forth. The SLP assessment setting was similar in this respect, but the focus here was on the child's language and communication, which is one of the main problems in a child with ASD and therefore may be somewhat easier to uncover by language assessment rather than by general developmental assessment. The ADOS and the medical evaluation are more unpredictable for the child, and both may require more flexibility on the part of the child. Given that negative reactions to new events and lack of flexibility are typical of autism, problems in these domains would perhaps be easier to detect than in a more structured environment.

Even though not significantly different in respect of diagnostic validity, preschool/home observation might be seen to be the most “informative” assessment setting of the six examined in this study. This does not mean to say that it is the “optimal” situation for the child, but it does provide the opportunity to see his/her functioning in everyday life. In the “free-field” preschool/home setting, the child's social communication problems become more obvious compared to typically developing peers. A proper assessment in this setting requires, of course, that the assessor has experience and knowledge of typical child development [13]. In Sweden, preschool groups of about 20 children are the rule; in this environment the child is expected to interact with other people, develop in play and everyday skills, and adapt to different routines, all of which tend to be difficult for a child with ASD. Therefore, it is not surprising that it was this kind of assessment that tended to lead to the best agreement with the final clinical diagnosis. In line with our findings, observation of young children in social settings has previously been suggested to be important [17]. 

The clinical impression and preliminary diagnostic assessment made after DISCO interview with a parent (before scoring for algorithm diagnosis) also corresponded quite well with final diagnosis. In particular “in the hands” of a medical doctor (who was “right” in 82% of the 17 cases she coded) with vast experience in the use of this particular instrument, the DISCO, to a very considerable degree, focuses on the parental experience of the child's functioning in everyday life. This further confirms earlier studies showing that information from parents and other caregivers is important in assessments of young children [17]. 

Assessment of the child's general development and cognitive ability is considered important in all neuropsychiatric assessments. On the basis of the present results we would argue that, in addition, it is important to observe the child in social, natural settings and that it is not sufficient to evaluate the child only in structured situations at the clinic. A previous study [27] showed high agreement between “textbook” ADOS observation at-the-clinic results and results obtained at preschool observation. When, as in the present study, clinical judgment is added in, one can speculate that the preschool observation of the child in interaction with his/her peers and the information from the preschool teachers together pick up the symptoms of ASD in a more meaningful way than the ADOS does. Observation of the child in the preschool can also provide information that can form the basis for an educational action plan and specific ASD appropriate interventions. This, of course, does not mean that ADOS is not an important instrument. Furthermore, some children do not attend preschool, or there could be other reasons why preschool observation cannot be made. Further clinical experience suggests that in some cases it may be crucial to include both preschool observation and clinic ADOS testing, for example, when ASD symptoms are less striking or when parents need “hands-on” information from watching the ADOS testing in order to better understand their child's problems. 

Despite the very limited number of girls in our study (*n* = 6), we found it to be of some interest that not one of the assessors estimated any of the girls as having greater problems than what the final diagnosis suggested. In addition, the assessment of diagnostic ASD status made immediately after DQ testing did not agree with final clinical diagnosis in any of the cases. If only one or two of the six different assessments had been used for assigning clinical ASD diagnoses, most of the girls in our study would have been “missed.” This should be a subject for further research as regards differences between girls and boys with suspected ASD. 

It is important to underline that the individual clinical assessment was made at the first meeting with the child. For some of the tests used (e.g., the DQ evaluation) the clinician would see the child more than once, and he/she might later receive additional information that could change the clinical appraisal of the child. We also have to consider that there could be a variation between the assessors with regard to being more or less “careful” to evaluate the degree of problems, when he/she saw the child for the first time. The fact that the children's problems varied with regard to DQ and autism symptoms and were more or less obvious at the first meeting with the child could also have affected the results. 

We are aware that there is a problem related to the confounding factor of the final comprehensive diagnosis being formulated on the basis of input from all the clinicians whose individual first impressions were then compared with the final diagnosis. However, none of the individual clinicians had the “final say” in respect of final diagnosis, and it would be clinically—and ethically—difficult, if not impossible, to design a study that would involve a completely independent comprehensive clinical diagnosis based on as many separate pieces of evidence which are needed when making a “comprehensive autism diagnosis based on all available evidence.” 

We used a rather small sample in our study, which reduces the possibility of generalization of our results to other clinical or community populations of preschool children. We also had a very small number of cases of children diagnosed with “no ASD,” limiting the possibility to conclude whether or not we can distinguish cases with milder symptoms of ASD from no ASD. Due to these limitations, it is important to consider the results cautiously and not to make too strong conclusions. However, we believe our findings add to those of earlier studies [12, 14], suggesting the view that clinical judgment is a crucial complement to formal tests. We also believe that it is important to make observations of the child in situations where the child's general behavior is as representative as possible. As emphasized by Huerta and Lord [17], multidisciplinary evaluations are important. 

Additional research with larger samples is needed in the field. We also propose that it is important to further examine how gender might possibly be important as a confounder in terms of getting a “correct” ASD diagnosis at young ages if children are assessed for ASD only by one or two examiners, however skilled they might be in diagnosing the disorder. As described in previous studies, stereotyped behaviors and so forth can be more obvious in children older than the participants in the current study [14], meaning that some symptoms might be easier to detect in some ages than in other ages. Multidisciplinary assessment by several team members would seem to be important when aiming to arrive at a valid clinical diagnosis. Interestingly, we found that one in seven to one in eight preschool children with a suspicion of ASD would be “misdiagnosed” if only the ADOS, the DISCO, or both had been used. 

Finally, we conclude that clinical ASD experience among assessing clinicians is of utmost importance. Extensive experience and good knowledge of typical development in young children would also be needed. 

### 4.1. Limitations

There are some obvious limitations, and, as previously mentioned, these need to be considered when interpreting our results. We had no comparison group, which means that we do not know how the results might have turned out if we included a more “mixed” sample. The sample was small, and, for this reason and because of the limitations previously discussed, the results have to be considered with caution. Also, within the context of the present study we could not perform a specific interrater reliability study across clinicians within the same professional category, for example, between the different education specialists. However, for the education specialists doing observations in preschool we have previously published the results of just such study [27], and we found that percent agreement ranged from 83% to 94% and weighted kappa from 0.82 to 0.93. In spite of these limitations, we do consider the results to be of interest for clinicians aiming to formulate guidelines for the diagnostic process for preschool children suspected of suffering from ASD. 

## Figures and Tables

**Table 1:** Sensitivity, specificity, and agreement of measurements between assessments and clinical diagnosis (all subjects).

Assessment	Sensitivity ASD clinical diagnosis (95% CI)	Specificity ASD clinical diagnosis (95% CI)	Weighted kappa (95% CI)	*n* (%) agreement	Clinical diagnosis more ASD	Clinical diagnosis less ASD	*P* value systematic changes between raters and clinical diagnosis
Medical/neurologic-psychiatric	0.64 (0.43; 0.82)	0.89 (0.52; 1.00)	0.40 (0.17–0.64)	21 (62%)	9 (26%)	4 (12%)	0.2668
DQ	0.40 (0.21; 0.61)	1.00 (0.66; 1.00)	0.28 (0.09–0.47)	16 (47%)	15 (44%)	3 (9%)	0.0075
Language	0.63 (0.41; 0.81)	0.78 (0.40; 0.97)	0.33 (0.08–0.59)	19 (58%)	9 (27%)	5 (15%)	0.4240
DISCO (parent interview)	0.74 (0.52; 0.90)	0.89 (0.52; 1.00)	0.40 (0.20–0.59)	20 (63 %)	7 (22%)	5 (16%)	0.7744
ADOS	0.60 (0.39; 0.79)	1.00 (0.66; 1.00)	0.43 (0.21–0.64)	21 (62%)	10 (29%)	3 (9%)	0.0923
Preschool/home	0.68 (0.46; 0.85)	0.89 (0.52; 1.00)	0.44 (0.18–0.69)	23 (68%)	8 (24%)	3 (9%)	0.2266

The sensitivity and specificity are calculated on the dichotomous variables ASD/no ASD, where “no ASD” also contains the classification “probably/possible ASD.” Weighted kappa, % agreement, clinical diagnosis more ASD, clinical diagnosis less ASD, and *P* value are all calculated at the 3-point division; ASD, ASD probable/possible; and no ASD.

**Table 2:** Individual assessor's agreement with final diagnosis.

Assessor	Same code as final diagnosis/number of assessments
Medical/neurologic-psychiatric	
1	14/20 (70%)
2	2/2 (100%)
3	1/3 (33%)
4	4/9 (44%)
DQ	
1	2/3 (67%)
2	3/9 (33%)
3	2/9 (22%)
4	9/13 (69%)
Language	
1	19/31 (61%)
2	0/2 (0%)
DISCO	
1	14/17 (82%)
2	2/6 (33%)
3	4/9 (44%)
ADOS	
1	8/15 (53%)
2	13/19 (69%)
Preschool/home	
1	11/19 (58%)
2	12/15 (80%)

One child did not have language evaluation, and 2 did not have the DISCO.
